# Differential effects of minified and magnified mirror visual feedback on the underlying misperception of hand size

**DOI:** 10.3758/s13414-021-02262-9

**Published:** 2021-03-22

**Authors:** A. Treshi-Marie Perera, Jiun Ting Tan, Poo Shin Mu, Roger Newport

**Affiliations:** 1 School of Psychology, University of Reading Malaysia, Iskandar Puteri, Malaysia; 2 Department of Psychology, Nottingham Trent University, 50 Shakespeare Street, Nottingham, UK; 3 School of Sport, Exercise and Health Sciences, Loughborough University, Epinal Way, Loughborough, UK

**Keywords:** Mirror visual feedback, Body representation, Hand size, Illusion susceptibility, Size perception

## Abstract

Perception of the size of body parts, for instance the hand, has been shown to be distorted in healthy participants, with over- and underestimations of width and length, respectively. Illusory manipulations of body shape and size have highlighted the flexibility of the body representation and have also been found to update immediate perceptions of body size and surrounding objects. Here, we examined whether underlying misperceptions of hand width and length can be modified through exposure to illusory changes in hand size using a mirror visual feedback (MVF) paradigm. While questionnaire responses indicated subjective susceptibility to both magnified and minified manipulations, objective hand size estimates only showed significant differences following exposure to minifying mirrors. These variations might reflect differences in the way that stored representations are accessed or updated in response to size manipulations. Secondly, the findings further reinforce differences between subjective and objective outcomes of illusions on subsequent body perception.

## Introduction

While it may seem intuitive that our somatic perceptions are hard-wired and resistant to manipulations, this is not always the case. In fact, numerous lines of clinical literature have provided evidence for altered bodily experiences, which can include vivid perceptions of amputated limbs (e.g. phantom limb syndrome; Ramachandran & Hirstein, 1998), misattribution of limbs to others (somatoparaphrenia; Vallar & Ronchi, 2009), and misperceptions of the shape and size of body parts as seen in patients with eating disorders and those suffering from forms of chronic pain (e.g., Gaudio & Quattrocchi, 2012; Gilpin, Moseley, Stanton, & Newport, 2014; Peltz et al., 2011). Interestingly however, distorted bodily experiences are not merely limited to clinical conditions, but are also fundamental characteristics of healthy body representations (Longo, 2017). For instance, Longo and Haggard (2010) identified systematic positional errors when healthy participants were asked to locate the position of a series of landmarks (fingertips and knuckles) on the dorsum of the hand. The nature of these was such that hand width is overestimated while hand length is underestimated, with the extent of underestimations increasing from the thumb to the little finger (Longo & Haggard, 2010). These findings suggest a distorted implicit representation of the body, which, in the case of the hand, appears *stubby* (Longo & Haggard, 2010). Subsequent research has demonstrated this effect to be stronger on the dorsum of the hand (compared to the palmer surface; Longo & Haggard, 2012a) and more pronounced on the hand than on non-corporeal objects, demonstrating these distortions to be hand-specific (Saulton, Dodds, Bülthoff, & de la Rosa, 2015). The functional mechanisms underlying such systematic distortions of the body representation are not fully understood, though it bears similarity to the distorted representation of body parts in the primary somatosensory cortex (S1; Penfield & Boldrey, 1937). This, together with the shape and organisation of the receptive fields of neurons representing the hand in S1, is thought to be linked to misperceptions of the metric properties of the body (or in this case, the hand; Longo, 2015; Longo & Haggard, 2010).

In parallel to these systematic misperceptions of hand width and length, the perceived size of equidistant tactile inputs is felt to be larger across the width of the hand than along the hand, suggesting a common body model to underlie both (Longo & Haggard, 2011). Moreover, in a perceptual phenomenon known as Weber’s illusion, tactile distances are perceived as being larger on more sensitive skin regions consisting of smaller and densely packed tactile receptive fields (i.e., the palm of the hand compared to the forearm; Weber, 1834/1996). Visual magnification and minification of the forearm and hand (respectively) nevertheless reverses this effect, suggesting that metric properties of touch are referred to the perceived size of body representations (Taylor-Clarke, Jacobsen, & Haggard, 2004; see also De Vignemont, Ehrsson, & Haggard, 2005). That tactile processing is altered as a result of manipulations to the perceived body representation therefore highlights the plasticity of implicit representations of the body.

Body shape and size perceptions are maintained by the integration of sensory signals including visual and proprioceptive inputs (Lackner, 1988; Taylor-Clarke et al., 2004); therefore, altering these inputs through illusory manipulations gives rise to changes in body size perception and highlights the dynamic flexibility of our body representation. In line with this, studies employing size-altering visuo-proprioceptive manipulations (Newport et al., 2015; Perera, Newport, & McKenzie, 2017) and mirror visual feedback (MVF) paradigms (Mancini, Longo, Kammers, & Haggard, 2011) have provided evidence for ownership over body parts of increased and decreased size, thus highlighting the bi-directional flexibility of our *own* body representation. In MVF paradigms, a vertical mirror is placed perpendicular to the body midline. Participants place one hand on either side, equidistant from the mirror, and lean over to one side slightly so that they can see into the mirror but cannot see the arm that is now behind the mirror. Thus, when they look across towards their hidden hand, they see a reflection of the non-hidden hand in exactly the same position (and posture) as the hidden hand. The visual appearance is one of looking at the hidden hand as if it can be seen directly. Evidence for ownership over magnified and minified reflections of the hand in MVF paradigms nevertheless remain inconsistent, with some studies only demonstrating weak ownership over reflections of altered hand size (Johnson & Gohil, 2016; Wittkopf, Lloyd, & Johnson, 2017). The reasons underlying such differences remain unclear but can be attributed to variations in experimental procedures such as the duration of mirror exposure and the presence/absence of visuomotor synchrony between the reflected hand and the hand placed behind the mirror (Holmes, Crozier, & Spence, 2004; Holmes & Spence, 2005; Medina, Khurana, & Coslett, 2015).

In addition to establishing ownership over manipulated representations of body parts, a few studies have also examined the extent to which such illusions update subsequent body part size judgements. For example, following a MVF paradigm that exposed participants to enlarged and reduced reflections of their hand, Mancini et al. (2011) found over- and underestimations of hand width, respectively, while Wittkopf et al. (2017) found underestimations in perceived hand length following exposure to mirror refelctions of reduced and normal hand size. Noteworthy, however, is that both studies compared perceived hand-size following exposure to the distorting mirror reflections with participants’ actual hand sizes, rather than to a pre-MVF baseline. Given the default misperceptions of implicit hand representations, the extent to which experimentally induced size manipulations alter these representations over and above our inherent propensities to misperceive hand width and length remains to be examined. Such an investigation would provide evidence for the stability and malleability of implicit body representations and be of relevance for clinical conditions characterised by body shape and size misperceptions, particularly when examining the effectiveness of illusion-based treatment interventions.

Using a MVF paradigm, the present study therefore aimed to examine the extent to which experimental manipulations (of hand size) altered subsequent hand size estimations whilst accounting for the ubiquitous misperceptions of hand size. As both hand width and length are subject to different size distortions (i.e., over- and underestimations, respectively), the effect of exposure to magnifying and minifying mirror reflections were separately examined for both. A previous study examining changes to perceived finger length before and after exposure to size altering visuo-proprioceptive illusions demonstrated length underestimations following the experience of reduced but not elongated manipulations (Perera et al., 2017). We therefore computed changes in hand width and length judgements following MVF exposure as a function of (pre-MVF) baseline hand size estimations and predicted significant underestimations in length and width following the minifying (but not magnifying) MVF conditions. These predictions were tested a priori using planned comparisons between magnifying/minifying and a normal (flat-mirror) condition. Given the inconsistencies in previous research, we also examined ownership over the magnified and minified mirror reflections of the hand. Whilst ownership over a normal-sized mirror reflection of the hand was expected, no directional predictions could be made about ownership over the magnified and minified reflections.

## Methods

### Participants

The study was approved by the University of Reading Malaysia Research Ethics Committee, and all participants gave written informed consent prior to participation. Eighty-six right-handed (Oldfield, 1971) participants were initially recruited. An a priori G*Power (version 3.1.9.3) analysis with the following parameters: effect size = 0.4, α = 0.05 and β = 0.80, recommended a sample size of 61 for χ^2^ tests. Additional participants were recruited to accommodate potential exclusions. Data of six participants were excluded from analyses as five were identified as outliers (with hand width/length estimates being 3 standard deviations away from the mean) and one participant withdrawing from the study. This brought the total sample size to 80 participants (26 males; age range 19–55 years; mean ± SD age 23.35 ± 6.84).

### Apparatus and procedure

Each participant was seated comfortably with their right hand resting on a table. They were then handed a block of Styrofoam and asked to estimate the length (distance from the tip of middle finger to wrist: *“Please estimate the length of your left hand, this is the distance from the tip of your middle finger to your wrist”*) and width (distance between knuckles of the little and index finger: *“Please estimate the width of your left hand now, this is the distance between your index and litter fingers”*) of their left hand by placing two drawing pins on it (referred to as *baseline* hand length/width estimates hereafter). Participants were instructed not to look at their left hand or use their other hand as a metric during hand length and width estimates. When needed, further gentle reminders were made to ensure that these instructions were adhered to. The orientation of the Styrofoam block (e.g., lateral/distal) and the order in which baseline length/width estimations were obtained was randomised across participants. Next, each participant went through the three mirror exposure conditions in a randomised order. Here, either a flat (no distortion), convex (magnifying) or concave (minifying) mirror mounted on a carboard box was placed in front of the participant such that the mirror was perpendicular to the body midline. The convex and concave mirrors had an approximate magnification and minification of ×2. The participant’s right and left hands (palm faced down) were positioned in front of and behind the mirror (i.e., inside the cardboard box), respectively, with the middle-fingers equidistant (20 cm) from the mirror. A black cloth placed over the participant’s left shoulder occluded its outline. The participant was then given the opportunity of acclimatising to the set-up and were instructed to carry out a series of synchronous hand movements (similar to those described in Wittkopf et al., 2017)^,^ which involved un/clenching fists and tapping their fingers (on the table) to the sound of a metronome. This took 3 min, after which the mirror was occluded, and participants were presented with an illusion questionnaire (see Table 1). These statements were presented in a randomised order on a laptop now placed to the participant’s right. The questionnaire examined ownership over the mirror reflected hand (statements 1–3, Table 1) and susceptibility to manipulations induced through the MVF paradigm (statements 4–6, Table 1) across the three conditions (flat-mirror, convex and concave). The questionnaire also controlled for task-compliance and suggestibility by including two control statements (statements 7 and 8, Table 1), and thus consisted of a total of eight statements. Participants responded on a 7-point Likert scale ranging from -3 (strongly disagree) to +3 (strongly agree), with 0 indicating neither agree nor disagree. Next, participants were again presented with a Styrofoam block and asked to place two drawing pins on it to estimate their hand length and width (referred to as post-illusion hand length/width estimates hereafter). Obtaining post-illusion hand size estimates followed the same procedure as the baseline estimates, and the order in which length/width estimates were obtained was randomised across participants. It should be noted that participants’ left hand was placed inside the mirror box (with the mirror occluded) and therefore out of view during post-illusion hand size estimation. As with the baseline hand size estimations, participants received explicit instructions and reminders that the right hand should not be used as a metric during these estimations. Four Styrofoam blocks of different lengths were prepared in advance to avoid participants’ hand length/width judgements being influenced by their previous estimates (including the baseline estimates). Finally, at the end of all three mirror exposure conditions, the participant’s actual hand length and width estimates was recorded.

**Table 1:** Statements in the illusion questionnaire

Statement number	Statement	Statement type
1	It seemed like I was looking directly at my hand rather at a reflection of the hand	Ownership
2	It seemed as if the reflection of the hand was my real hand	
3	It seemed as if the reflection of my hand was part of my body	
4	It seemed as if my left hand was in the same location as the reflection of the hand	Illusion susceptibility
5	My left hand seemed larger than normal	
6	My left hand seemed smaller than normal	
7	It seemed like I had two left hands	Control
8	It seemed as though I no longer had a left hand	

## Results

### Responses to questionnaire items

Ratings to the ownership statements were separately averaged to compute an overall score for each MVF condition. The effect of condition on ownership was then examined by comparing ownership scores across the three MVF conditions with a Friedman ANOVA. Further Wilcoxon signed-rank tests comparing overall ownership scores to each control statement score established the presence of ownership in each condition and confirmed that these effects were not influenced by suggestibility or task-demand effects. Next, to ascertain that the MVF paradigm did indeed give rise to convincing manipulations, statements 4, 5 and 6 were also separately compared across conditions with Friedman ANOVAs. Finally, one-sample signs tests were conducted on statements 1–6 to establish that these statements were in fact greater than 0 (neither agree nor disagree). Effect sizes reported were computed using the following formula: r = Z /*(√n).*

### Ownership

**Fig. 1:**
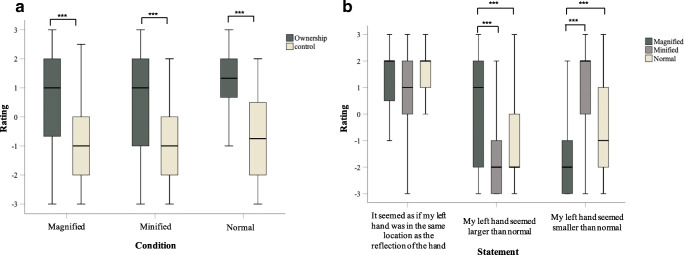
Box and whisker plots for (**a**) overall ownership and control ratings (**b**) ratings to the mirror visual feedback susceptibility items across the three conditions (*** *p* ≤ 0.001)

A Friedman ANOVA indicated no difference in overall illusion scores across all three conditions (χ^2^ [2, N = 80] = 2.44, *p* = 0.30), suggesting no difference in the extent to which participants were susceptible to the minified, magnified and normal mirror conditions. Overall illusion scores were also significantly higher and positive (i.e., all medians ≥ 1) compared to the control scores (all medians = -1) in all conditions (see Fig. 1a); magnified (*Z* = 6.34, *p* < 0.001; r = 0.71), minified (*Z* = 5.93, *p* < 0.001; r = 0.66) and normal (Z = 6.92, p < 0.001; r = 0.77)^1^. Bonferroni corrections adjusting the significance value to *p* = 0.017 (*p*/3) were applied to account for multiple comparisons.

Furthermore, Bonferroni-corrected one-sample sign tests (adjusted *p* = 0.008; *p*/6) indicated that all ownership scores were significantly greater than 0 (all *p* ≤ 0.004), and thus further affirmed ownership over the mirror reflected hand across all conditions. Ratings to control statements across the three conditions were significantly less than 0 (all *p* ≤ 0.002; Bonferroni-corrected significance level = 0.017).

### Illusion susceptibility

Next, ratings to statements 4 (*“It seemed as if my left hand was in the same location as the reflection of the hand”*), 5 (*“My left hand seemed larger than normal”*) and 6 (*“My left hand seemed smaller than normal”*) were separately compared across the three MVF conditions.

A Friedman ANOVA conducted on statement 4 revealed no significant main effect (χ^2^ [2, N = 80] = 3.50, *p* = 0.17; all medians ≥ 1), indicating that the reflected hand was perceived to be in the same location as the real hand in all conditions (see Fig. 1b). Ratings to statement 4 were also significantly greater than 0 as indicated by one-sample sign tests (all *p* ≤ 0.001).

A significant main effect of condition was observed for statement 5 (χ^2^ [2, N = 80] = 68.96, *p* < 0.001), with participants agreeing that their left hand felt significantly larger following the magnified condition (median = 1) compared to the minified (*Z* = 6.61, *p* < 0.001; r = 0.74; median = -2) and normal (*Z* = 5.79, *p* < 0.001; r = 0.65; median = -2). Bonferroni corrections adjusting the significance value to *p* = 0.025 (*p*/2) were applied.

Similarly, a main effect of condition was also observed for statement 6 (χ^2^ [2, N = 80] = 70.53, *p* < 0.001), with participants agreeing that their hand felt smaller following the minified condition (median = 2) compared to the magnified (*Z* =- 6.65, *p* < 0.001; r = 0.74; median = -2) and normal (*Z* = 5.20, *p* < 0.001; r = 0.58; median = -1) conditions. Bonferroni corrections adjusting the significance value to *p* = 0.025 (*p*/2) were made. One-sample sign tests indicated that ratings to statements 5 and 6 were significantly greater than 0 in both the magnified and the minified conditions, respectively (all *p* ≤ 0.004 at Bonferroni-corrected levels of *p* = 0.017), suggesting that participants strongly experienced their perceived hand size to be manipulated (see Fig. 1b).

### Hand-size judgements

Baseline hand length and width estimates were first compared to actual hand length and width. Next, percent over-and underestimations of *baseline* hand size were obtained by calculating the difference between baseline and actual hand size calculated as the percentage of actual hand size, for both length and width separately. Similarly, percent changes in hand size estimation following each MVF condition was also computed by calculating the difference between post-illusion and baseline hand size as the percentage of baseline hand size. Positive and negative values indicated over- and underestimations, respectively. Participants’ actual hand width was not normally distributed (Shapiro-Wilk <0.05). Percent over- and underestimation of hand width and length was also not normally distributed in some conditions (Shapiro-Wilk <0.05), therefore non-parametric analyses were conducted. Friedman tests were used to compare differences in post-illusion hand size estimations across the three MVF conditions and followed up with planned Wilcoxon signed-rank tests for length and width independently.

### Estimations of baseline hand-size

**Fig. 2:**
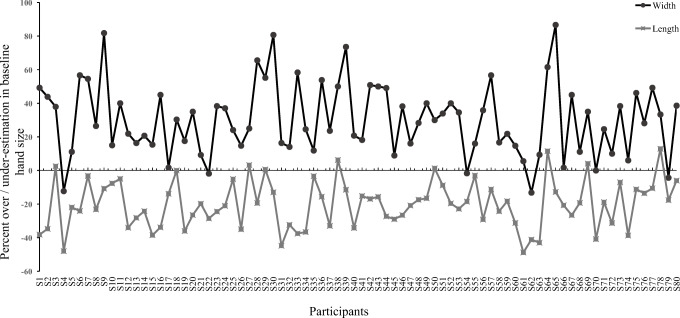
Percent over- and underestimations of veridical hand width and length for all participants

Compared to actual hand width and length, baseline hand size showed underestimations in length (*Z* = -7.37, *p* < .001; r = 0.82) and overestimations in width (*Z* = -7.52, *p* <.001; r = 0.84). Percent underestimation of veridical hand length was 19.71%, while hand width demonstrated an overestimation of 28.21%. Figure 2 demonstrates the patterns of over- and underestimations of baseline hand width and length computed as a function of actual hand width and length for all participants.

### Post-illusion hand-size estimations

#### Length

**Fig. 3:**
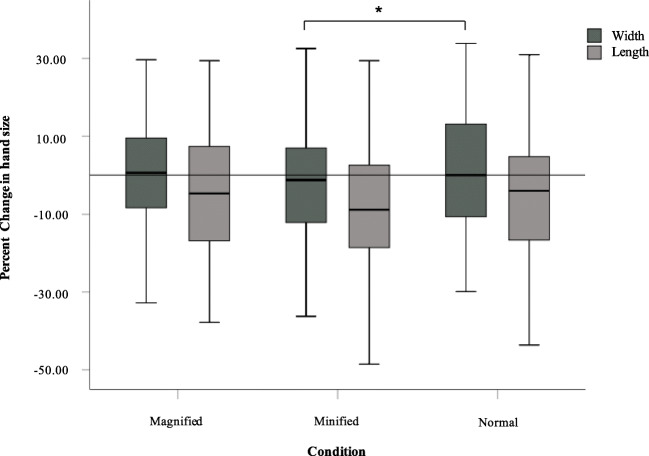
Percent change in hand width and length judgements across the three conditions as a function of baseline hand size estimations (* *p* < 0.05)

A Friedman ANOVA conducted to examine changes in percent differences in hand length estimates post-illusion revealed a significant main effect (﻿χ^2^ = [2, N=80] = 8.31, *p* = 0.016; see Fig. 3). Planned comparisons revealed a trend towards under-estimations in hand length following minified condition compared to the normal (*Z* = -1.94, *p* = 0.053; r = 0.23) but no difference between the magnified and normal condition (*Z* = -1.07, *p* = 0.28).

#### Width

A Friedman ANOVA conducted to examine changes in percent differences in hand-width estimates following the MVF conditions also revealed a significant main effect (χ^2^ = [2, N=80] = 8.98, *p* = 0.011; see Fig. 3). Planned comparisons indicated underestimation of hand width in the minified condition compared to normal (*Z* = -2.11, *p* = 0.035; r = 0.24), but no difference between the magnified compared to normal conditions (*Z* = -0.22, *p* = 0 .82).

## Discussion

Using a mirror visual feedback paradigm our results demonstrated that perceived size of the hand can be modified without loss of ownership. Such modifications were nevertheless restricted to underestimations of hand size following minified mirror exposure. These changes altered metrics in hand width (and marginally in length) and were additional to the underlying distortions of hand size.

### MVF manipulation strength and ownership

Subjective responses indicated that our MVF paradigm successfully updated the perceived size of participants’ hand and that participants experienced the reflection of their hand to be in the same location of their actual hand. Importantly, no significant differences in ownership were observed across the three MVF conditions, indicating that participants felt they were looking at their own hand irrespective of the size manipulations. It can hence be concluded that synchronous visuomotor stimulation of both hands induced feelings that the seen hand was part of their own body irrespective of its size. That subjective perceptions of hand size can be manipulated while preserving ownership highlights the malleability of our body representation (e.g., Normand, Giannopoulos, Spanlang, & Slater, 2011; van der Hoort et al., 2011; Perera et al., 2017) and extends previous studies that have found voluntary movements to enable self-recognition (e.g., Newport, Pearce, & Preston, 2010; Tsakiris, Prabhu, & Haggard, 2006) even in the presence of distortions (Ratcliffe & Newport, 2017). These findings contradict some early rubber/fake hand illusion paradigms that have demonstrated asymmetric tendencies to incorporate only enlarged body parts to our body representation (Pavani & Zampini, 2007; although see also van der Hoort et al., 2011). Indeed, engaging in self-generated active movements, as opposed to receiving passive stimulation on the seen and unseen hands, might have induced a sense of agency (Kilteni et al., 2012; Perera et al., 2017) over the mirror reflected hand across all MVF conditions, which could have facilitated ownership and the experience that the reflected left hand was part of the body. As such our findings may also highlight limitations when employing purely passive multisensory paradigms when investigating the interplay between mechanisms of sensory integration and body ownership.

### Hand size judgements

In line with the findings of Longo and Haggard (2010), we found underlying over- and underestimations of hand width and length, respectively, suggesting that healthy representations of our body size are distorted. Such misperceptions of size have been attributed to the ovular shape and proximodistal orientation of somatosensory receptive fields on the back of the hand (Alloway, Rosenthal, & Burton, 1989; Brooks, Rudomin, & Slayman, 1961; Longo & Haggard, 2011). It should, however, be noted that while qualitatively similar, the magnitude of veridical hand width and length misperceptions in the current study was smaller compared to that initially reported by Longo and Haggard (2010). In the present study, we found hand width and length over- and underestimations of approximately 30% and 20%, respectively, whereas hand width and finger length over- and underestimations initially reported by Longo and Haggard (2010) were 70% and 28%, respectively. Such quantitative differences in misperceptions of hand width and length are indeed apparent throughout similar studies (e.g., Coelho & Gonzalez, 2019), and in light of the present experiment demonstrates that directional misperceptions of hand size can be replicated across different methodologies. Importantly, our results also demonstrated these default misperceptions to be updated following exposure to minified (but not magnified) manipulations of hand size in the direction of the manipulation. Interestingly, hand length underestimations persisted even following magnifying manipulations, whilst the ratio of baseline hand width and length misperceptions were preserved even following minifying manipulations. Therefore, whilst previous research (e.g., Mancini et al., 2011; Wittkopf et al., 2017) has demonstrated experimental manipulations of size to update perceived hand length and width, such observations may only be apparent when making comparisons against actual hand size, rather than against the perceived (and distorted) mental representation of the hand.

Given our constant exposure to body parts growing in size (De Vignemont et al., 2005), underestimations in hand size following brief exposures to minifying experimental manipulations may seem counterintuitive, hence here we attempt to discuss potential mechanisms that might underlie this observation. Carruthers (2008) distinguished between two body representations, an online representation constructed from incoming sensory information that reflects current perceptions of the body (i.e., what the body is like now), and an offline representation that reflects what the body is usually like and is primarily constructed from online representations (although see also Tsakiris & Fotopoulou, 2008). In the current experimental paradigm, participants made judgements of body size either pre- or post-exposure to the illusory manipulations and not *during* exposure to the manipulated mirror visual feedback. Therefore, as opposed to being influenced by current incoming sensory information, post-illusion hand size estimates might have been tempered by stored offline body representations. Indeed, stored offline body representations have previously been shown to be more resistant to enlarging illusions (Perera et al., 2017), thus preventing further changes to perceived hand size. One explanation here could be that cortical representations that specify body shape and size across development (Melzack, Israel, Lacroix, & Schultz, 1997; O’Shaughnessy, 1995) could operate compensatory mechanisms preventing further overestimations of body size (e.g., Taylor-Clarke et al., 2004). Indeed, despite the disproportionate representation of the hands along the somatosensory and motor cortices (Penfield & Boldrey, 1937), no corresponding biases in actions or tactile processing are observed, which suggests the presence of compensatory mechanisms correcting for these over-representations (Cardinali, Gori, & Serino, 2019; Longo & Haggard, 2011; Taylor-Clarke et al., 2004). This serves as an explanation underlying underestimations in hand size; based on this, we therefore hypothesise that any further experimental manipulations of increased size could operate stronger compensatory mechanisms thus preventing overestimations in hand size following magnification. Alternatively, if as suggested by Carruthers (2008), offline representations of the body are constructed from online representations, the brief exposure to magnified illusory manipulations would have perhaps not been sufficient to update or lead to an overestimation of body size (for the hand at least). Therefore, long-term (offline) representations of the body in the brain might only be readily malleable in one direction – minification. This, nevertheless, warrants further investigation.

Moreover, our results also indicated differences between the subjective and behavioural measures of body size perception. While participants seemed to strongly experience that larger and smaller representations of the hands were a part of their body at a subjective level, no concordant updating of the body representation was observed at a behavioural level following exposure to magnified representation. This is potentially due to both subjective illusion experiences and the behavioural body size estimation methods, tapping into different representations of the body. While subjective responses might have reflected immediate online perceptions of the body based on current sensory input during exposure to the mirror feedback manipulations, this might not have been the case during subsequent hand width and length estimates in the behavioural task. Previous research has indeed demonstrated perceived hand size to be updated in the direction of both enlarged/shrunken manipulations with online but not offline body size estimation tasks (Perera et al., 2017). Additionally, these findings could also represent the dissociations between taxonomies of body representations – the body image and body schema. Accordingly, our subjective and behavioural body size assessments would have recruited these representations to varying extents. The body image is a conscious representation of the body’s form/appearance, which is based on at least three aspects: the body’s perceptual properties (i.e., how we perceive the body), our cognitive attitudes towards the body (or knowledge about the body) and how we feel about our body (Gallagher, 1986). In relation to this, participants’ subjective responses reflect perceptual aspects of the body image that was readily updated through synchronous visuomotor feedback (e.g., Kammers, de Vignemont, Verhagen, & Dijkerman, 2009; Newport et al., 2010). The body schema on the other hand, is dynamic, unconscious and represents the body’s spatial properties (Gallagher, 1986), including the shape and configuration of body segments in space (Haggard & Wolpert, 2005). As such, the post-MVF hand size estimations would have been more strongly weighted on the body schema, thus suggesting that unconscious body representations may not be as readily updated following sensorimotor feedback. Supporting this claim, some research has demonstrated different methods of measuring body size to differently influence subsequent body size perceptions (Cash & Deagle, 1997; Longo & Haggard, 2012b).

In their localisation task, Longo and Haggard (2010, 2012a, b) demonstrated distortions in veridical hand length and width judgements when participants marked the position of a series of landmarks on an occluded hand through a screen. On the one hand the present size estimation task shares similarity to the localisation task in that participants were estimating the *position* of two points of their hand, we also acknowledge differences between the two tasks. As previously stated, despite differences in magnitude, however, our body size estimation task also demonstrated distortions analogous to the localisation task, and thus suggests that perhaps a common body representation might underlie both the positional errors of hand size estimates (Longo & Haggard, 2010, 2012a, b) and the explicit hand size misperceptions in the current study (Longo, 2017). Accounting for such qualitatively similar misperceptions of the body representation across various measurement methods, Longo (2015) suggested that body representations might perhaps exist along a continuum extending from primary somatotopic maps representing the body to conscious perceptions of our body (Longo, 2015, 2017). Bodily perceptions may therefore be a result of the relative weighting placed on either side of the continuum (Longo, 2015). Such a model might not only account for the qualitative similarities in our behavioural body size estimation task and the localisation task (Longo & Haggard, 2010, 2012a, b), but perhaps also for differences in response patterns across our subjective and behavioural tasks.

In conclusion, this study demonstrates that both perceived length and width judgements are updated following exposure to certain experiential manipulations of the hand – minification. As opposed to comparing changes in perceived hand size to actual hand size, the present study provides evidence for an updated hand size perception whilst factoring the inherent distortions already present in hand size estimation. Therefore, misperceptions of our veridical body size estimates (e.g., Longo & Haggard, 2010), while malleable, might be unidirectional. Whether or not such underestimations following illusory manipulations are only specific to the body representation nevertheless warrants further investigation. Finally, we also showed differences in subjective illusion susceptibility and behavioural body size estimates, which might suggest that the two measures might tap into separate online and offline body representations.

None of the data or materials for the experiments reported here is available, and none of the experiments was preregistered.

## Data Availability

The dataset generated during and/or analysed during the study are available from the corresponding author on reasonable request.

## Footnotes

1 As the two control statements reflected different experiences – the presence of an additional hand and the absence of a hand – we also compared ownership with the two control items (statements 7 and 8) separately. Here again, a similar pattern of results emerged, with ownership being significantly higher and positive compared to control statements 7 (median = -1) and 8 (median = -1) across all conditions (all *p* < 0.001).
